# Alpha-Synuclein in Parkinson's Disease: From Pathogenetic Dysfunction to Potential Clinical Application

**DOI:** 10.1155/2016/1720621

**Published:** 2016-08-17

**Authors:** Lingjia Xu, Jiali Pu

**Affiliations:** Department of Neurology, 2nd Affiliated Hospital, School of Medicine, Zhejiang University, Hangzhou, Zhejiang 310009, China

## Abstract

Parkinson's disease is a neurodegenerative disease/synucleinopathy that develops slowly; however, there is no efficient method of early diagnosis, nor is there a cure. Progressive dopaminergic neuronal cell loss in the substantia nigra pars compacta and widespread aggregation of the *α*-synuclein protein (encoded by the* SNCA* gene) in the form of Lewy bodies and Lewy neurites are the neuropathological hallmarks of Parkinson's disease. The* SNCA* gene has undergone gene duplications, triplications, and point mutations. However, the specific mechanism of *α*-synuclein in Parkinson's disease remains obscure. Recent research showed that various *α*-synuclein oligomers, pathological aggregation, and propagation appear to be harmful in certain areas in Parkinson's disease patients. This review summarizes our current knowledge of the pathogenetic dysfunction of *α*-synuclein associated with Parkinson's disease and highlights current approaches that seek to develop this protein as a possible diagnostic biomarker and therapeutic target.

## 1. Introduction

Parkinson's disease (PD) is the second most common neurodegenerative disorder [1] and is defined as one of the synucleinopathies, which include other disorders featuring Lewy bodies [2]. It is characterized by the relatively selective loss of dopaminergic neuronal cells in the substantia nigra pars compacta (SNpc) and the presence of Lewy bodies and Lewy neurites in surviving affected neurons [3]. As the main component of the Lewy bodies and Lewy neurites, *α*-synuclein is the product of the first gene identified as associated with PD:* SNAC*, which was reported in 1997 by Polymeropoulos et al. [4]. Mutations in* SNCA* (duplications, triplications, or point mutation) cause autosomal dominant forms of PD and are the basis of the risk of developing sporadic PD [5]. Recent studies [6–8] suggested that the misfolding of *α*-synuclein causes it to aggregate and spread in certain sites, where the inflammation induced by it is intimately involved in the pathogenetic dysfunction underlying PD. All this indicates that *α*-synuclein plays a central role in the pathogenesis of PD.

Currently, the main treatment for PD is replacement therapy using levodopa, which may be effective in the early stage of the disease [9]. However, as the disease progresses, levodopa has less effect, and a series of side effects, such as movement complications, occur. Therefore, other therapeutic strategies, such as deep brain stimulation (DBS), have also been attempted for advanced patients; however, it is only an alleviative treatment. Consequently, biomarkers for early diagnosis and neuroprotective therapy are urgently required for this chronic disorder. Alpha-synuclein is the distinctive hallmark of PD; therefore, it has a potential application in the clinical diagnosis and treatment of PD [10].

To fully understand the pathogenetic dysfunction of *α*-synuclein associated with PD, in this review, we summarize the current knowledge of the physiology and pathology of *α*-synuclein, including its structure, physiological function, degradation, spread, and toxicity. We also highlight current approaches that seek to develop this protein as a potential diagnostic biomarker and therapeutic target.

## 2. Alpha-Synuclein Structure and Physiological Function

In humans, *α*-synuclein is a member of a three-protein family: *α*-synuclein, *β*-synuclein, and *γ*-synuclein [11]. Alpha-synuclein is a small protein comprising 140 amino acids with three domains: an N-terminal domain (aa 1–65), a non-amyloid-*β* component of plaques (NAC) domain (aa 66–95), and a C-terminal domain (aa 96–140) [12]. Rare point mutations in the N-terminal domain of *α*-synuclein, such as Ala53Thr, Ala30Pro, Glu46Lys, and the recently described His50Gln, Gly51Asp, and Ala53Glu, result in autosomal dominant familial PD and PD-like syndromes, presumably caused by misfolding and/or aggregation of the mutant *α*-synuclein protein [4, 13–17]. All known clinical mutations are present in this N-terminal region [10], emphasizing the importance of this domain in the pathological dysfunction of *α*-synuclein. The NAC domain, which is unique to *α*-synuclein [18], has a stretch of 12 amino acid residues that are responsible for the aggregation properties of *α*-synuclein via inhibition of its degradation and promotion of its fibrillation [19]. Nowadays, most studies focus on the N-terminal peptide; however, future studies should also consider the C-terminal peptide, because this is where truncation more typically occurs [20]. The truncations discovered to date include Tyr39T, Tyr125T, Tyr133T, and Tyr136T [10]. To date, very few studies have investigated the effects of the smallest peptide produced by truncation. Research on this peptide might give us a new and distinct view of the potential application of this protein.

Concerning the native state of *α*-synuclein, there are two hypotheses: one is the monomeric conformation, and the other one is the *α*-helically folded tetramer. Early studies of *α*-synuclein isolated from bacterial expression systems or mouse tissues indicated that it is monomeric, with a limited secondary structure [21]; however, Bartels et al. [22] identified the state of endogenous *α*-synuclein in living human cells by examining freshly collected human red blood cells and showed that natively, endogenous cellular *α*-synuclein exists largely as an *α*-helically folded, 58 kDa tetramer. They hypothesized that the contrasting results might have resulted from the different materials and protocols applied in this research, namely, denaturing detergents. The tetramer circulates in plasma and can become destabilized which promotes *α*-synuclein aggregation from monomers to oligomers. Further studies by Burré et al. [23], using similar methods in the mouse brain, indicated that the predominant native conformation of *α*-synuclein might be an unstructured monomer, exhibiting a random coil structure in solution, and it can aggregate age-dependently, while the *α*-helical structure was only adopted upon membrane binding [24].

However, the normal physiological structure and function of *α*-synuclein still remain unclear.

Recent studies showed that the normal physiological function of *α*-synuclein involves roles in compartmentalization, storage, and recycling of neurotransmitters [25]. In addition, *α*-synuclein is associated with the physiological regulation of certain enzymes and is thought to increase the number of dopamine transporter molecules [26]. Neurotransmitter release [27] and interaction with the synaptic SNARE- (soluble N-ethylmaleimide-sensitive factor attachment protein receptors) complex are partly mediated by its role as molecular chaperone [23]. Cycling between SNARE-complex assembly and disassembly is required, with continuous generation of reactive SNARE-protein intermediates. Cysteine string protein *α* (CSP*α*) is a chaperone that is essential for synaptic health, whose deletion in mice led to a decrease in the SNARE-complex, nerve terminal degeneration, motor impairment, and cell death [28]. In CSP*α* knockout mice, *α*-synuclein could rescue this degenerative phenotype and restore levels of SNARE-complexes in synaptic terminals. Moreover, mice lacking both *α*-synuclein and CSP*α* exhibited nerve terminal dysfunction and cell death [29]. These findings suggested that *α*-synuclein is able to complement the activity of CSP*α* as a molecular chaperone. This interaction was documented in further research in which *α*-synuclein was demonstrated to directly bind to the SNARE-protein synaptobrevin-2 and promote SNARE-complex via binding of its C-terminal 44 residues to the N-terminal 28 residues from synaptobrevin-2 [30].

## 3. Alpha-Synuclein Aggregation, Degradation, and Spread

**Figure 1:**
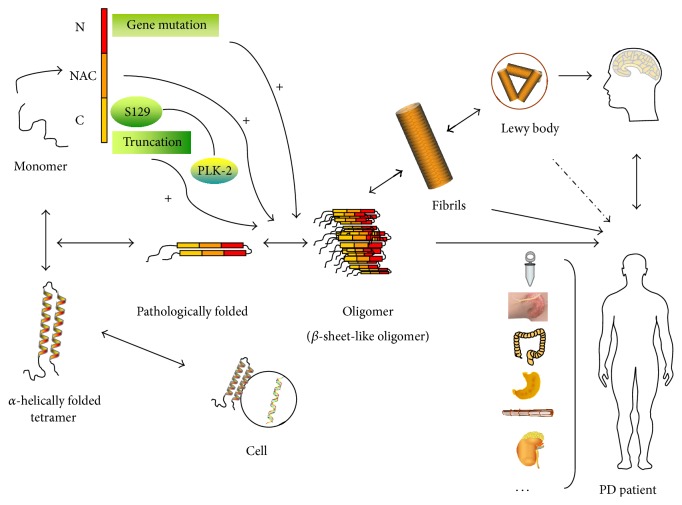
Alpha-synuclein's aggregation pathway and role as a diagnostic biomarker in PD. Alpha-synuclein is a small protein comprising 140 amino acids with three domains that exist in dynamic states. The *α*-helically folded tetramer is thought to be only adopted upon membrane binding. The three domains each have a role in aggregation, as shown in the figure. All known gene mutations are found in the N-terminal domain, and some have been proven to accelerate aggregation. The NAC domain has a stretch of 12 amino acid residues that are unique and typical in the formation of oligomers and fibrils. C-terminally truncated *α*-synuclein appears to aggregate faster. In addition, the phosphorylation of amino acid 129, located in C-terminal domain, plays a central role in the pathway and is promoted by PLK2. Alpha-synuclein leads to toxicity when aggregated into pathological oligomers, fibrils, and Lewy bodies. In the search for a diagnostic biomarker in PD, *α*-synuclein from the CSF, plasma, the submandibular gland, saliva, colonic and gastric mucosa samples, and peripheral nerve fibers has been tested.

Alpha-synuclein exists in various conformations in a dynamic equilibrium, modulated by many factors, comprising internal and external factors that either accelerate or inhibit fibrillation [31–33]. As mentioned before, disease-related mutations affect the aggregation of *α*-synuclein (Figure 1). All known mutations associated with familial PD (Ala53Thr, Ala30Pro, Glu46Lys, His50Gln, Gly51Asp, and Ala53Glu) are found in the N-terminal domain [10]. The mutations Glu46Lys, His50Gln, and Ala53Glu [14, 15, 17] can promote *α*-synuclein to form insoluble aggregates and produce oligomers. However, how these mutations accelerate aggregation has not been completely clarified. Based on Burré et al.'s later study [30], it is likely to be due to the destabilization of the native N-terminal conformation. The NAC domain plays a central role in *α*-synuclein's self-propagation [19]. Recently, Rodriguez et al. [34] resolved the crystal structures of residues 68–78 (termed NACore) and residues 47–56 (PreNAC) using microelectron diffraction, which revealed that, in certain regions, these strands transferring into *β*-sheets are typical of amyloid assemblies. Lastly, the C-terminal domain was identified to be necessary to maintain the solubility of *α*-synuclein. The presence of residues consisting of five prolines suggested that this region lacks secondary structure [35]. In addition, C-terminally truncated forms of *α*-synuclein appeared to aggregate faster than the full-length protein [36, 37]. In addition, the C-terminus appears to be important for the interaction of *α*-synuclein with other proteins in the nervous system and with some small molecules [23]. These findings indicated that all three domains play a role in aggregation and that they might influence each other, either promoting or inhibiting its pathological fibrillation and oligomerization.

Phosphorylation of *α*-synuclein is essential and sufficient in the process of degradation in neurodegenerative diseases. Mass methodologies revealed that *α*-synuclein extracted from human Lewy bodies was phosphorylated at S129 [38]. Some data indicated that polo-like kinase (PLK) 2-mediated phosphorylation of S129 increased autophagy-mediated degradation of *α*-synuclein, suggesting that phosphorylation might be a neuroprotective mechanism to accelerate the clearance of aggregated protein [39]. Chemical nitration of *α*-synuclein resulted in the formation of both tyrosine-nitrated monomers and nitrated dimers [40], which also affected the degradation of *α*-synuclein, and immunoelectron microscopy confirmed that nitrated monomers and dimers are incorporated into amyloid fibrils. Purified nitrated *α*-synuclein monomer by itself was unable to form fibrils, whereas the nitrated dimer accelerated the aggregation of unmodified *α*-synuclein [41]. Additionally, nitration of certain residues in the N-terminal domain decreased binding to synthetic vesicles and prevented the protein from adopting the *α*-helical conformation to the membrane [41]. The structure has been identified by Snead and Eliezer [42], clarifying the physiological function of *α*-synuclein binding to the membrane. Using the synthetic nitrated *α*-synuclein, the results showed that nitration did not interfere with phosphorylation of S129 by* PLK3* and reaffirmed that intermolecular interactions between the N- and C-terminal domains of *α*-synuclein are critical to direct nitration-induced oligomerization of *α*-synuclein [30].

Alpha-synuclein is degraded by the ubiquitin-proteasome system (UPS) and the autophagy-lysosomal pathway [43]. Ebrahimi-Fakhari et al. [44] provided* in vivo* evidence that normal soluble *α*-synuclein is degraded mainly by the UPS, whereas more complex conformations, including aggregates, are degraded by the autophagy-lysosomal pathway. The finding that *α*-synuclein in both the monomeric and oligomeric states can be detected in human plasma, cerebrospinal fluid, and other peripheral tissues [45, 46] suggested the idea that *α*-synuclein is secreted. Although the exact mechanism of *α*-synuclein's release has not been fully demonstrated, it seems that *α*-synuclein might be released by exosomes in a calcium-dependent manner and be further degraded after lysosomal inhibition [47]. Lööv et al. [48] found that insoluble conformations of *α*-synuclein do not themselves appear to have significant neurotoxic effects, despite being misfolded and even aggregated in certain areas. By contrast, various *α*-synuclein oligomers are harmful, and structures termed extracellular vesicles (EVs) might mediate the propagation of toxic *α*-synuclein between neurons [48]. In one study, recombinant *α*-synuclein monomers produced together with EV fractions from cultured neuroblastoma cells accelerated the formation of toxic oligomers compared with monomeric *α*-synuclein produced alone [49]. EVs are mediators of cellular information; thus, genetic information can be carried from one cell to another and consequently can aggravate the toxicity [50]. In conclusion, propagation and spreading are key to the pathogenetic dysfunction in PD. Recent* in vivo* and* in vitro* studies [26] confirmed that transfer and interaction through the membranes by *α*-synuclein might contribute to the pathogenetic dysfunction in PD and thus progress the disease. These results suggest that *α*-synuclein propagation is a major factor in the progression of PD pathology.

Moreover, numerous data have suggested that *α*-synuclein self-propagates [7]. Normally, small numbers of aggregates are disposed of by the protein degradation pathways; however, if, over time, the aggregates accumulate above a certain threshold, they could self-propagate, contributing to the progression of PD. Lewy bodies and neurites, a histopathological signature of PD, found in grafted fetal dopaminergic neurons in the SNpc of PD patients, are of significant importance [51]. These observations led to the development of the “prion-like hypothesis” [51]. Several* in vitro* and* in vivo* studies suggested that *α*-synuclein can spread from cell to cell and from region to region, which dramatically promotes PD pathogenesis and progression [52–58]. Recently, one of these studies focused on the postmortem analyses of brains from patients with PD who received fetal mesencephalic transplants and demonstrated that *α*-synuclein-containing Lewy bodies gradually appeared in the grafted neurons [53]. Subsequently, The authors in [53] seeded *α*-synuclein aggregates in recipient neurons to explore whether intercellular transfer of *α*-synuclein could occur from the host to the graft. Ultimately, they demonstrated that *α*-synuclein could transfer between host cells and grafted dopaminergic neurons. In summary, intercellularly transferred *α*-synuclein can propagate its pathology by interacting with cytoplasmic *α*-synuclein. However, whether the pathological conversion of endogenous *α*-synuclein is triggered by material derived from patients with PD or from recombinant *α*-synuclein remains to be discussed. In addition, whether preformed fibrils might occur directly through a seeding prion process or occur indirectly as a general response to cellular stress remains unknown.

## 4. Alpha-Synuclein Toxicity in PD

The precise mechanism whereby *α*-synuclein leads to toxicity and cell death remains obscure. It is likely that aggregation of *α*-synuclein results either from an increased release of *α*-synuclein and increased cell-to-cell transfer or via accumulated cellular levels of the protein [38]. Here, we discuss the latest research in this area.

Alpha-synuclein's toxicity is interconnected with its physiological function, and to better understand its toxicity, animal models, including wild-type ones and those with genetic mutations, are needed. One of the most important physiological functions that *α*-synuclein regulates, synaptic activity, was tested directly in mice lacking *α*-synuclein. Originally, *α*-synuclein null mice develop normal brain architecture and contacts and do not exhibit gross behavioral phenotypes [59]. Upon repeated stimulation, dopaminergic synapses from *α*-synuclein null mice showed highly elevated dopamine release [59]. In *α*/*β*-synuclein double knockout mice, synaptic plasticity appears unaltered relative to *α*-synuclein single knockouts, although the dopamine levels in the striatum were reduced [60]. Meanwhile, in the *α*/*β*/*γ*-synuclein triple knockout mice, the synucleins were proved to be very important, because of the decreased life span and age-dependently synaptic dysfunction compared with wild-type mice [23]. Collectively, these reports emphasized the important role of the synucleins in long-term synaptic maintenance and flexibility. Kokhan et al. [61] carried out behavioral evaluations in *α*-synuclein knockout mice, and the results showed that *α*-synuclein knockout mice had worse learning ability in tests requiring both working and spatial memory. For the first time, they demonstrated that *α*-synuclein is necessary for these types of learning and explained this phenomenon by discussing neurotransmitters involved in the pathology of cognitive dysfunction, like monoamine, glutamate, and acetylcholine-mediated neurotransmission [61]. The physiological function and pathological dysfunction of *α*-synuclein are both involved in synaptic neuronal transmitters, which prompts the question as to what triggers this protein's toxicity.

The neuronal toxicities of *α*-synuclein caused by genetic mutations or epigenetic mechanisms appear to involve many pathways and cellular functions, including endocytosis, Golgi homeostasis, ER-to-Golgi transport, presynaptic trafficking, UPS, autophagy, ER, and oxidative and nitration stress [62–64]. Alpha-synuclein oligomers are thought to be the toxic species and the cause of the neurodegenerative process. These oligomers would spread throughout the brain and other parts of body and induce *α*-synuclein pathology in interconnected structures [48].

There are several pathological factors that contribute to the toxicity of *α*-synuclein. Firstly, dysfunction of autophagy and UPS, the two main ways to clear toxic *α*-synuclein [65, 66], might lead to neuronal toxicities; secondly, both nitration and oxidation decrease the propensity of *α*-synuclein to form stable conformations, which might contribute to the progression of PD; in addition, truncated *α*-synuclein species have also been reported in Lewy bodies [20]. Truncation, typically occurring in the C-terminal domain of the protein, is associated with an increased propensity of *α*-synuclein to form fibrils and with increased toxicity in fly and rat models of PD [67, 68]. Currently, inflammation is a hot topic in studies of the pathogenesis in PD. Glial cells are the culprit in the mechanism of neuroinflammation, and this makes sense considering the prion-like hypothesis of *α*-synuclein's spread throughout the brain. The direct transfer of *α*-synuclein from neurons to astrocytes was demonstrated* in vivo* using transgenic mice overexpressing human *α*-synuclein under a neuronal promoter by Lee et al. [69]. In these transgenic mice, accumulation of human *α*-synuclein was observed not only in neurons, but also in glial cells [69]; the authors also found that the secretion of *α*-synuclein by neurons induced toxicity not only inside the cytoplasm of neighboring cells, but also in the extracellular space. The results clarified what activates glial cells and induces chronic inflammation, thereby contributing to the progression of the pathology throughout the brain. In other reports, the preferential binding of iron, copper, and other metals, including Cu(II), Mn(II), Co(II), and Ni(II), to the C-terminus of *α*-synuclein at residues D121, N122, and E123 [70, 71] has been shown to influence *α*-synuclein's function and aggregation and to promote the disease.

The spread of *α*-synuclein suggests that its toxicity would affect both the nervous system and other systems throughout the human body. This prompted us to consider the relationship between *α*-synuclein and the nonmotor symptoms in PD, such as the deficit of the olfactory sensation and astriction, which are nonspecific and always appear before the motor symptoms. Olfactory filaments are the only nerves directly exposed to the exterior environment [10, 56]. Transgenic animals expressing human *α*-synuclein under the control of the tyrosine hydroxylase promoter (ensuring catecholaminergic neuron-specific expression) presented olfactory impairments compared with wild-type animals during the olfaction test, and the olfactory deficits appeared long before the motor alterations in that study [52]. This brain region is of particular interest, because Lewy neurites and bodies are present in this area in the very early stages of PD [52]. This also provided a new insight into the toxicity of *α*-synuclein and its potential as a biomarker. However, it remains to be determined whether the misfolding of *α*-synuclein occurs randomly, where and when it first appears, and how self-propagation is initiated.

## 5. Alpha-Synuclein as a Diagnostic Biomarker in PD

To date, the diagnosis of PD still relies mostly on clinical features, because neuropathological confirmation is only possible with autopsy examination in postmortem studies [72]. Early diagnosis is required urgently, since PET-CT (Positron Emission Computed Tomography) or functional MRI (Magnetic Resonance Imaging) scans are not specific enough for this disease. Alpha-synuclein, with its unique characteristics in the occurrence and development of synucleinopathies, exists widely, not only in the central nervous system, but also in the peripheral nervous system, submandibular gland, skin, and saliva gland [72], making it a good candidate as a diagnostic biomarker, especially at the early stage of the disease.

About five years ago, studies provided evidence that *α*-synuclein was present in the CSF from PD patients [73]; however, the role of *α*-synuclein species in PD prognosis remains unclear [74]. Subsequently, some studies tested the level of *α*-synuclein in plasma after controlling several major variables; however, unlike CSF, there were no obvious differences between PD patients and controls. Recently, the submandibular gland was shown to be involved in synucleinopathy in the early stages of PD [75]. Consequently, Devic et al. [76] investigated human saliva, and the results seemed positive, suggesting that saliva *α*-synuclein is another potential biomarker for PD's diagnosis and progression. Recently, the presence of *α*-synuclein reactive antibodies in the serum of PD patients has become a hot topic [77].

New evidence has emerged indicating that CNS-derived EVs in plasma could serve as diagnostic biomarkers [48]. In addition, other studies have shown that urine harbors EVs; therefore, if the EVs could be isolated successfully, urine would be another example of an easily accessible biofluid [78]. Hypothetically, in addition to testing for *α*-synuclein itself, the whole structure that generated, transported, and even cleared *α*-synuclein could be detected. Zange et al. [79] tested skin from 10 patients with multiple system atrophy and 10 with PD together with six control subjects suffering from essential tremor; the phosphorylated *α*-synuclein in the specimens was examined by immunohistochemistry, and both phosphorylated *α*-synuclein deposits in skin sympathetic nerve fibers and dermal nerve fiber density were assessed. Their results showed that all patients with PD expressed phosphorylated *α*-synuclein in sympathetic skin nerve fibers, correlating with age-independent denervation of autonomic skin elements. In contrast, no phosphorylated *α*-synuclein was found in patients with multiple system atrophy or in the essential tremor-control subjects. These findings supported the view that phosphorylated *α*-synuclein deposition may cause nerve fiber degeneration in PD. Although the peripheral synuclein tissue is a closer step to diagnosis of PD, Tolosa and Vilas [80] pointed out that Miki et al. [81] and Navarro-Otano et al. [82] made efforts to find abnormal *α*-synuclein deposition in the gastrointestinal tract and failed. Afterwards, several studies have identified phosphorylated *α*-synuclein in gastric and colonic specimens, as well as in the salivary glands. However, there are still some important methodological issues that need to be discussed. Firstly, the optimal site of *α*-synuclein deposits in skin has not yet been identified and current evidence suggests it might occur in skin tissue obtained from the cervical region [83]. Secondly, the number of biopsies needed to obtain a convincing result is also unclear. Thus, further studies are needed to determine the sensitivity and specificity of *α*-synuclein as a diagnostic biomarker for PD. Eventually, studies targeting testing phosphorylated *α*-synuclein in the peripheral nervous system in PD are still desperately needed [80]. Studies that aimed to achieve pathological confirmation of PD by biopsying these accessible tissues or chemical examinations evaluating the levels of *α*-synuclein are summarized in Table 1 [45, 46, 73–77, 83–91]. Currently, the nonmotor symptoms are becoming more and more important in the diagnosis of PD; however, they are always nonspecific and easily ignored by the patients. If physicians could find successfully a way to identify the close relationship between synucleins and the pathology of PD, great progress in the early and differential diagnosis of PD would be made. In addition, there have been few studies targeting synuclein using magnetic resonance or PET; therefore, more research effort is required.

## 6. Alpha-Synuclein as a Therapeutic Target in PD

There are four common ways to combat the toxicity produced by *α*-synuclein: decrease *α*-synuclein aggregation, control its propagation, increase its clearance, and stabilize its existing circumstances. A correct protein balance has a central role in cellular homeostasis of the nervous system [92].

Many mediators participate in the neurotoxicity induced by *α*-synuclein in synucleinopathies. For example, the inflammatory protease caspase-1 mediates the C-terminal truncation and was implicated in the mechanism in promoting aggregation of *α*-synuclein* in vitro* and* in vivo *[20]. Interestingly, a caspase-1 inhibitor could provide neuroprotective effects on PD by reducing *α*-synuclein cleavage, hence limiting its ability to form aggregates. Preventing aggregation could also be achieved using passive or active immunization approaches, such as gene-silence technologies or active protein immunization. There are already some transgenic mouse models of PD reported [93] that have reached the clinical investigation stage.

Dehay et al. aimed to prevent either direct *α*-synuclein's seeds' toxicity or cell-to-cell transmission and have developed some* in vitro* screens for compounds targeting these phenomena [94]. Models with human Lewy body-derived *α*-synuclein assemblies can also be used to prevent cell-to-cell transmission. As discussed above, the spread of *α*-synuclein includes neuron to neuron, neuron to glia, glia to neuron, and glia to glia [69]. A combination of these methods would allow the identification of potential therapeutics.

The two major degradation systems are autophagy and the UPS. The UPS is thought to be responsible for the degradation of misfolded proteins [95]. A study aimed at this system indicated that downregulation of the UPS might contribute to the pathogenesis of PD [66]. Moreover, considering neurodegenerative diseases, aging is the most significant risk factor for the development of such diseases and is associated with progressive decline of the UPS and accumulation of oxidized proteins [96]. This suggests that targeting these two systems to increase the clearance of *α*-synuclein might be an efficient treatment for PD in the future.

Many studies have reported the development of powerful tools and models targeting *α*-synuclein. In addition, much attention is now being paid to the proteotoxic mechanisms and inflammation induced by *α*-synuclein and how to block them using strategies such as enhancing cellular clearance through innate and adaptive immunization [25]. The accumulation of C-terminal domain truncated *α*-synuclein can be inhibited by immunotherapy [8]. In addition, improvements in axonal and motor deficits can be achieved by protecting C-terminal domain truncated *α*-synuclein from C-terminal cleavage [68]. Furthermore, the antibodies that inhibit C-terminal truncation could, theoretically, reduce cell-to-cell propagation of *α*-synuclein. Immunization with antibodies targeting the C-terminal truncation sites of *α*-synuclein, the oxidation and nitration of *α*-synuclein, or even those promoting increased clearance might have therapeutic potential, not only as agents to reduce the amount of *α*-synuclein itself, but also as inhibitors of its pathological oligomerization and propagation. Several important questions concerning the antibodies remain, the most fundamental one being how antibodies could reach the brain compartment at sufficient levels and how they could recognize their intracellular targeting protein and promote its intracellular toxicity.

Small molecules that stabilize *α*-synuclein's physiological tetramer could reduce its pathogenicity. The JAK/STAT (Janus kinase/signal transducer and activator of transcription) pathway is known to function in cell proliferation, differentiation, and apoptosis and in immune regulation and hematopoietic cells generation and plays a variety of biological functions in tumorigenesis and neural development. Cytokines such as interleukin, interferon, and epidermal growth factor can contribute to the protection of the nervous system through this pathway, which also provided new insights into the future therapy of PD [97].

## 7. Conclusion

Alpha-synuclein is a major component of Lewy bodies and Lewy neurites, which are the neuropathological hallmarks of Parkinson's disease. Currently, gene-targeting therapy and biotherapy are hot topics in research into neurodegenerative disorders such as Parkinson's disease, Alzheimer's disease, and Huntington's disease. Here, we summarized recent progress targeting this unique protein. However, further research effort is required and several questions remain: What is the specific mechanism of this protein in PD? Do other, as yet undiscovered, gene mutations or duplications or triplications lead to the production of the toxic version of this protein? Did the gene mutations initiate its dysfunction? How can we control the toxic effects of this protein if we aim to limit the accumulation of misfolded proteins without disturbing its physiological function? In conclusion, we still lack critical knowledge necessary to develop *α*-synuclein as a diagnostic biomarker and therapeutic target.

## Figures and Tables

**Table 1:** Selected studies targeting *α*-synuclein as biomarker for the diagnosis of PD.

Ref	Materials	Analytical/measuring methods	Results in PD patients compared to controls
Lebouvier et al., 2008 [84]	Colonic tissue	Biopsy and immunohistochemical studies	TH-IR (tyrosine-hydroxylase immunoreactive) neurons were not a marker but phospho-*α*-synuclein-IR neurities were found in PD patients
Beach et al., 2010 [75]	Lower esophagus and submandibular tissue	Biopsy and a sensitive immunohistochemical method for phosphorylated *α*-synuclein	A rostrocaudal gradient of decreasing phosphorylated *α*-synuclein histopathology frequency and density
Shi et al., 2010 [45]	Alpha-synuclein in plasma	Blood component separation and analysis	No statistical difference was observed
Cersósimo et al., 2011 [85]	Salivary gland	Biopsy and immunohistochemical studies	The presence of *α*-synuclein inclusions in the submandibular glands
Devic et al., 2011 [76]	Saliva	Immunoblotting with a rabbit anti-human *α*-synuclein antibody ASY-1	The level of *α*-synuclein decreased
Yanamandra et al., 2011 [77]	Alpha-synuclein reactive antibodies in blood *sera*	ELISA, western blot, and Biacore surface plasmon resonance	Higher antibody levels towards monomeric *α*-synuclein
Shannon et al., 2012 [86]	Colonic submucosa	Biopsy and immunohistochemical studies	Staining for *α*-synuclein in nerve fibers in colonic submucosa
Alexoudi et al., 2013 [87]	Submandibular gland	Topic discussion	Positive
Schmid et al., 2013 [88]	Alpha-synuclein posttranslational modifications (PTMs)	A new chemical synthesis scheme	Relevant PTMs associated with disease progression and severity
Adler et al., 2014 [89]	Submandibular gland	Biopsy and immunohistochemical studies	Microscopic evidence of the tissue was positive for Lewy type *α*-synucleinopathy
Gao et al., 2015 [46]	CSF	Meta-analysis	The mean CSF *α*-synuclein concentration was significantly lower
Sanchez-Ferro et al., 2015 [90]	Gastric mucosa samples	Biopsy and immunohistochemical studies	Positive fibers for the *α*-synuclein protein were observed
Zhou et al., 2015 [73]	CSF	Meta-analysis	Mean concentration of CSF *α*-synuclein was slightly decreased; mean concentration of CSF *α*-synuclein oligomers was significantly higher
Adler et al., 2016 [91]	Submandibular gland	Biopsy and immunohistochemical studies	Positive staining
Donadio et al., 2016 [83]	Skin nerve	Skin biopsy	Only 49% of samples with a higher positivity rate for abnormal *α*-synuclein deposits at the proximal site in IPD
Parnetti et al., 2016 [74]	CSF	Review of 32 selected articles	The role of *α*-synuclein species in PD prognosis remained unsatisfactory
